# Associations among telomerase activity, p53 protein overexpression, and genetic instability in lung cancer

**DOI:** 10.1038/sj.bjc.6690378

**Published:** 1999-05-01

**Authors:** X Wu, B Kemp, C I Amos, S E Honn, W Zhang, G L Walsh, M R Spitz

**Affiliations:** Departments of Epidemiology, Pathology, Neurooncology, andThoracic and Cardiovascular Surgery, The University of Texas MD Anderson Cancer Center, 1515 Holcombe Blvd, Houston, TX 77030, USA; School of Public Health, The University of Texas Health Science Center at Houston, Houston, TX, USA

**Keywords:** telomerase activity, p53, genetic instability, lung cancer

## Abstract

Genomic instability is a driving force for tumorigenesis. p53 and telomerase play central roles in maintaining genomic integrity. The purpose of this study was to assess the associations among p53 protein overexpression, telomerase activity and genetic instability in lung cancer. We found that telomerase activity was detectable in 80% of 100 lung tumours, but only 7.7% of 91 paired adjacent normal tissues. p53 protein was overexpressed in 63% of the tumours but only 2% of the normal tissues. p53 was overexpressed in 56 of the 80 (70%) tumour tissues with telomerase activity but only seven of the 20 (35%) without telomerase activity. p53 protein overexpression carried a 6.7-fold (95% confidence interval, 1.7–27.7) increased risk for positive telomerase activity after adjustment by age, sex, ethnicity, smoking status and family history of lung cancer. The mean in vitro bleomycin-induced breaks per cell (a marker of cancer susceptibility) was significantly higher (0.92) for patients who overexpressed p53 in lung tumour tissue than that for patients with no detectable p53 expression in lung tumour tissue (0.65). Our data suggest that p53 protein overexpression may be common in individuals genetically susceptible to carcinogen exposure. p53 status may be related to telomerase expression. © 1999 Cancer Research Campaign


*British Journal of Cancer* (1999) **80**(3/4), 453–457
© 1999 Cancer Research Campaign
Article no. bjoc.1998.0378

### Associations among telomerase activity, p53 protein overexpression, and genetic instability in lung cancer

**X Wu[1,5], B Kemp[2], CI Amos[1], SE Honn[1], W Zhang[3], GL Walsh[4] and MR Spitz[1]**

Departments of [1]Epidemiology, [2]Pathology, [3]Neurooncology and [4]Thoracic and Cardiovascular Surgery, The University of Texas MD Anderson Cancer Center, 1515 Holcombe Blvd, Houston, TX 77030, USA; [5]School of Public Health, The University of Texas Health Science Center at Houston, Houston, TX, USA

**Summary** Genomic instability is a driving force for tumorigenesis. p53 and telomerase play central roles in maintaining genomic integrity. The purpose of this study was to assess the associations among p53 protein overexpression, telomerase activity and genetic instability in lung cancer. We found that telomerase activity was detectable in 80% of 100 lung tumours, but only 7.7% of 91 paired adjacent normal tissues. p53 protein was overexpressed in 63% of the tumours but only 2% of the normal tissues. p53 was overexpressed in 56 of the 80 (70%) tumour tissues with telomerase activity but only seven of the 20 (35%) without telomerase activity. p53 protein overexpression carried a 6.7-fold (95% confidence interval, 1.7–27.7) increased risk for positive telomerase activity after adjustment by age, sex, ethnicity, smoking status and family history of lung cancer. The mean in vitro bleomycin-induced breaks per cell (a marker of cancer susceptibility) was significantly higher (0.92) for patients who overexpressed p53 in lung tumour tissue than that for patients with no detectable p53 expression in lung tumour tissue (0.65). Our data suggest that p53 protein overexpression may be common in individuals genetically susceptible to carcinogen exposure. p53 status may be related to telomerase expression.

**Keywords:** telomerase activity; p53; genetic instability; lung cancer

Genomic instability reflects the propensity and susceptibility of the genome for acquiring multiple alterations and, in turn, is believed to be a driving force behind multistep carcinogenesis. Hsu et al (1983) have hypothesized that constitutional genetic instability is not an all-or-none phenomenon but instead exists in varying degrees in the general population, with one extreme end of the spectrum being the chromosome instability syndromes. Genetic instability can be unmasked by mutagen challenge in vitro. The mutagen sensitivity assay, which quantifies in vitro bleomycin-induced chromatid breaks in short-term cultured lymphocytes, was developed as a measure of constitutional genetic instability (the net result of DNA repair capability and initial genetic instability) (Hsu et al, 1989). This notion was supported by the evidence that mutagen sensitivity is an independent cancer risk predictor (Spitz et al, 1989, 1994; Strom et al 1995; Wu et al, 1995*a*, 1995*b*, 1996) and is not modulated by age, gender, smoking status, or tumour burden (Wu et al, 1995*b*).

p53 is believed to play a central role in maintaining genomic stability (Marx, 1994). Mutation of p53 is one of the most frequent genetic alterations in solid tumours (Hollstein et al, 1991). Telomerase activity and immortalization have also been implicated in tumorigenesis. Telomeres are the TTAGGG repeats at the physical ends of eukaryotic chromosomes. The function of telomeres is to prevent chromosomes from degrading and fusing with each other. Each cell division shortens the telomeres. Telomerase is an enzyme that can add telomeric sequences to the ends of chromosome to compensate for the losses that occur with each round of DNA replication (Harley and Villeponteau, 1995). Telomerase activity is present in germline cells, but somatic cells do not have telomerase activity and stop dividing when their telomeres have been shortened to a critical length. Telomerase expression may be related to immortalization (Kim et al, 1994). Human cancer cells, in post-mortality stage I (M1) and pre-mortality stage II (M2) are mortal cells without telomerase activity. Cancer cells that pass M2 are immortal cells with telomerase activity (Wright et al, 1989; Shay et al, 1991). It has been proposed that one of the main functions of p53 may be to detect telomere erosion and subsequently signal growth control pathways (Wynford-Thomas et al, 1995).

As stated above, p53 and telomerase activity associated with genomic integrity and p53 may play a role in tumorigenesis through telomere erosion detection. Furthermore, a significant correlation between p53 oncoprotein overexpression and mutagen sensitivity in head and neck cancer patients with multiple malignancies has been reported (Gallo et al, 1995). We hypothesized that p53 protein overexpression and the presence of telomerase activity would be more common in individuals with genetic susceptibility to carcinogenic exposure than in those individuals without genetic susceptibility and p53 protein overexpression might be correlated with the presence of telomerase activity in lung tumorigenesis. The purpose of this study was to assess this hypothesis in paired lung tumour tissue and peripheral blood lymphocytes (PBLs).

*Received 27 January 1998*
*Revised 11 September 1998*
*Accepted 20 October 1998*

*Correspondence to:* X Wu, Department of Epidemiology, Box 189, The University of Texas MD Anderson Cancer Center, 1515 Holcombe Blvd., Houston, TX 77030, USA

### MATERIALS AND METHODS

### Samples and subjects

We collected 100 fresh lung tumour tissues at thoracotomy and 91 paired adjacent normal tissues from patients with non-small cell lung cancer who underwent surgical treatment at M. D. Anderson

Cancer Center from 1993 to 1997. The patients were 59 men, 41 women, 88 whites, seven Mexican Americans, four African Americans, and one Asian. Their ages ranged from 20 to 80 years, with a mean age of 63.8 years. The specimens were stored immediately after excision at –80°C until they were subjected to the telomeric repeat amplification protocol (TRAP) and Western blotting assays. The clinical and epidemiological data were derived from chart review. Ten-millilitre paired PBLs samples were obtained from a subset ($n$ = 44) of these patients.

### Protein extraction

Fifty to 150 mg of frozen tissue was washed in a lysis buffer three times and then homogenized in 100 μl of precooled lysis buffer. After 30 min of incubation on ice, the lysate was centrifuged at 14 000 rpm for 20 min at 4°C, and the resulting supernatant was rapidly frozen at –80°C. The concentration of protein in each extract was measured using the bovine serum albumin (BSA) protein assay kit.

### Measurement of telomerase activity

Telomerase activity was measured by the highly sensitive polymerase chain reaction (PCR)-based TRAP method with an internal telomerase assay standard (Piatyszek et al, 1995). The assay was performed in a 50-μl reaction mixture containing 6 μg of protein extract, 50 μM dNTP, 0.1 μg of the deoxyoligonucleotide primer TS (5′-AATCCGTCGAGCAGAGTT-3′), 1 μg of T4 gene 32 protein, 5 ag of internal telomerase assay standard (a 150-bp cDNA fragment), 24 Ci $mmol^{-1}$ [α-$^{32}$P]-dCTP, and 2 U of *Taq* polymerase in a 0.5-ml tube that contained 0.1 μg of the deoxyoligonucleotide CX (5′-CCCTTACCCTTACCCTTACCCTAA-3′) sequestered at the bottom by a wax barrier. After 30 min of incubation at room temperature, which allowed telomerase-mediated extension of the TS primer, the reaction mixture was heated at 90°C for 90 s to inactivate the telomerase and then at 94°C for 35 s to denature the DNA. The reaction mixture was then subjected to 30 PCR cycles of 94°C for 30 s, 50°C for 30 s and 72°C for 45 s, and a final extension step of 72°C for 60 s. The PCR product was subjected to electrophoresis in a 10% acrylamide gel, which was then autoradiographed.

Extracts from tissues not containing telomerase did not extend the TS primer. A sample was classified as telomerase-positive if it had an RNAase-sensitive 6-bp DNA ladder. An internal control was used to identify false negative samples that contained *Taq* polymerase inhibitors. Some such samples gave false negative results with the standard 6 μg of protein extract per assay but positive results when diluted tenfold to 100-fold. Therefore, the telomerase activities of samples yielding negative results and no internal control signals were estimated by serial dilution of the sample. An RNAase-treated sample and lysis buffer were used as a negative control.

### Measurement of p53 expression

Aliquots of extract containing 40 μg of protein were used for measuring p53 levels. The extracted protein was analysed on a sodium dodecyl sulphate (SDS)-polyacrylamide gel as described previously (Zhang et al, 1995). After transfer to an Immobilon membrane, the protein was incubated overnight with antibody against p53 (Ab-6; Oncogene Science, Inc., Uniondale, NY, USA). The levels of protein were analysed using the enhanced chemiluminescence system (Amersham Corp., Arlington Heights, IL, USA) according to the manufacturer's instructions.

**Table 1** Host characteristics, telomerase activity and p53 expression in lung tumour tissue[a]

| | Total | Adenocarcinoma | Squamous carcinoma | Others[b] |
|---|---|---|---|---|
| Sex | | | | |
| Male | 59 (59.0) | 27 (50.0) | 23 (67.6) | 9 (75.0) |
| Female | 41 (41.0) | 27 (50.0) | 11 (32.4) | 3 (25.0) |
| Ethnicity | | | | |
| Non-Hispanic white | 88 (88.0) | 49 (90.7) | 28 (82.3) | 11 (91.7) |
| Hispanic | 7 (7.0) | 3 (5.6) | 4 (11.8) | 0 (0.0) |
| Black | 4 (4.0) | 2 (3.7) | 2 (5.9) | 0 (0.0) |
| Asian | 1 (1.0) | 0 (0.0) | 0 (0.0) | 1 (8.3) |
| Mean age | | | | |
| Years (s.d.) | 63.8 (11.3) | 62.9 (11.1) | 65.9 (8.6) | 60.1 (17.7) |
| Smoking status | | | | |
| Never | 10 (11.6) | 7 (14.9) | 1 (3.3) | 2 (22.2) |
| Ever | 76 (88.4) | 40 (85.1) | 29 (96.7) | 7 (77.8) |
| Tumour stage | | | | |
| I | 39 (41.5) | 20 (37.0) | 19 (59.4) | 0 (0.0) |
| II | 12 (12.8) | 7 (13.0) | 4 (12.5) | 1 (12.5) |
| III | 33 (35.1) | 20 (37.0) | 8 (25.0) | 5 (62.5) |
| IV | 10 (10.6) | 7 (13.0) | 1 (3.1) | 2 (25.0) |
| Family history of cancer | | | | |
| Positive | 52 (58.4) | 28 (56.0) | 17 (56.7) | 7 (77.8) |
| Negative | 37 (41.6) | 22 (44.0) | 13 (43.3) | 2 (22.2) |
| p53 overexpression | | | | |
| Positive | 63 (63.0) | 36 (66.7) | 19 (55.9) | 8 (66.7) |
| Negative | 37 (37.0) | 18 (33.3) | 15 (44.1) | 4 (33.3) |
| Telomerase activity | | | | |
| Positive | 80 (80.0) | 43 (79.6) | 27 (79.4) | 10 (83.3) |
| Negative | 20 (20.0) | 11 (20.4) | 7 (20.6) | 2 (16.7) |

[a]Number (%) except as indicated. [b]Includes larger cell carcinoma, non-differentiated non-small-cell carcinoma, bronchioalveolar carcinoma, and tumours of unknown histologic type.

### Mutagen sensitivity assay

Genetic instability was measured by the mutagen sensitivity assay, which has been described in detail previously (Hsu et al, 1989). Briefly, 1 ml of peripheral blood was added to 9 ml of RPMI-1640 supplemented with 20% fetal bovine serum, 2 mM L-glutamine, 50 U $ml^{-1}$ penicillin, 100 μg $ml^{-1}$ streptomycin and 1.3% phytohaemagglutinin. After 67 h of incubation, the cultures were treated with bleomycin (0.03 U $ml^{-1}$) for 5 h. At 72 h, the cells were treated with colcemid (0.04 μg $ml^{-1}$) to arrest the cells in mitosis. Harvesting, fixation, slide preparation and staining were carried out in a standard way. Fifty metaphases per sample from the coded slides were read to count the number of chromatid breaks. Mutagen sensitivity was expressed as the average number of breaks per cell. We recorded only frank chromatid breaks or exchanges, and disregarded chromatid gaps or attenuated regions.

### Statistical analysis

Positive telomerase activity was defined as the presence of an RNAase sensitive 6-bp DNA ladder. p53 protein overexpression was defined as the presence of a band under the exposure condition we used. Mutagen sensitivity was expressed as the number of induced chromatid breaks per cell from scoring 50 metaphases. Kendall's Tau-b correlation coefficient was used to characterize

© Cancer Research Campaign 1999

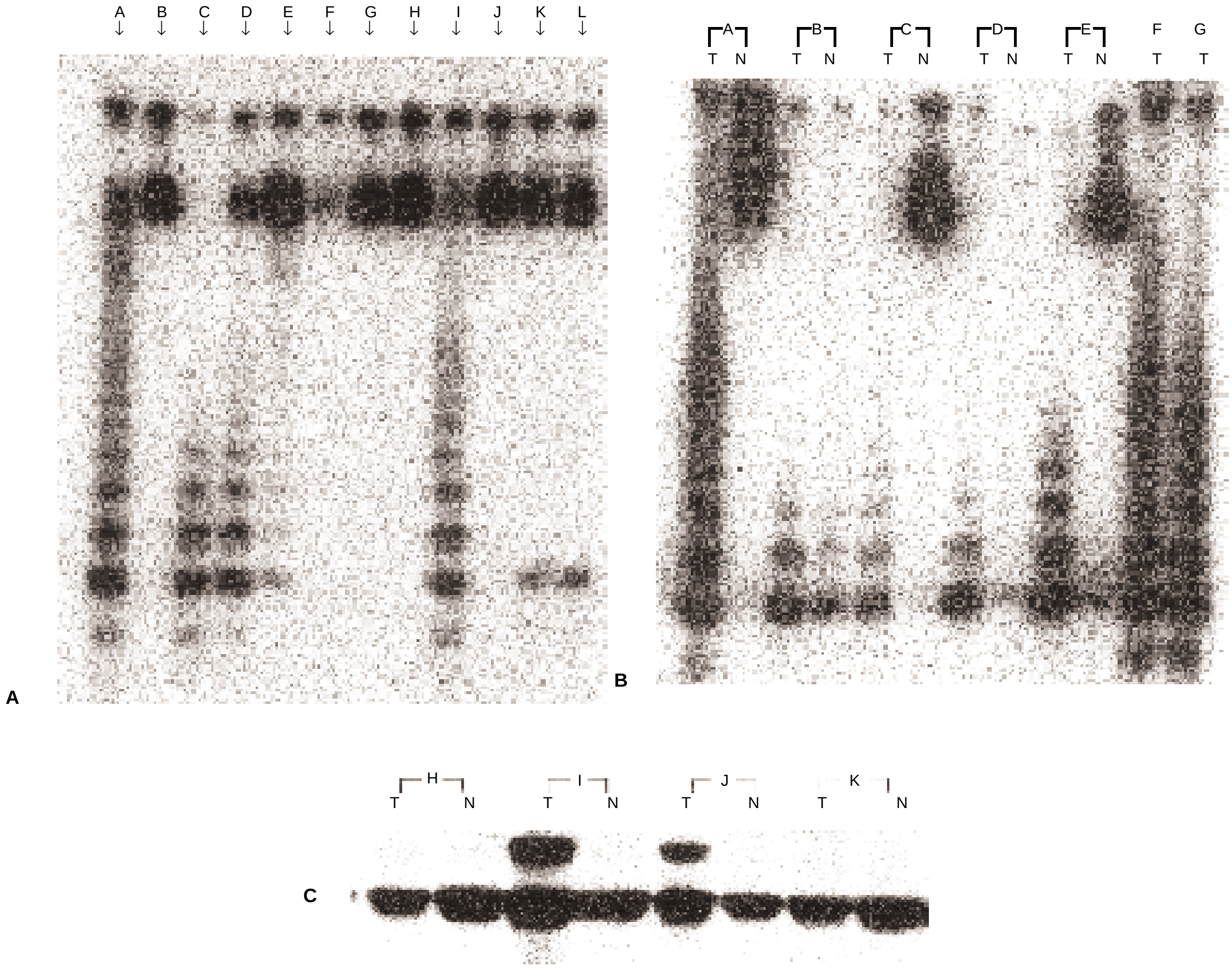

**Figure 1** **(A)** Measurement of telomerase activity. Lane A, a positive control with 6-bp ladder signals; lane B, a negative control with lysis buffer; lanes C–E, serial dilution (6 μg, 0.6 μg, 0.06 μg) of extract from one subject (all dilutions are positive); lane F, negative signal with the weak internal control signal suggesting the presents of polymerase inhibitor; lanes G–H, tenfold and 100-fold dilutions of sample in lane F; lane I, a case with positive telomerase activity; lane J, sample in lane I pretreated with RNAase, which abolished telomerase activity and provided a control for the specificity of the assay; lane K, sample with one band that may reflect telomerase activity or a primer dimer; lane L, sample in lane K treated with RNAase, which did not abolish the band indicating that the sample was telomerase negative. **(B)** Various levels of telomerase activity in tumour tissues and adjacent normal tissues. Tumour samples from cases A, B, C, D, E, F and G and normal tissue of case B showed positive 6-bp ladder signals. The normal samples of cases A, C, D and E were negative. **(C)** Various levels of p53 protein expression in tumour tissues and adjacent normal tissues. Tumour samples from cases I and J showed p53 overexpression. Cases H and K showed no detectable p53 protein expression. Normal tissue from cases H, I, J and K showed no detectable p53 protein expression the correlation between p53 overexpression and telomerase activity. Student's *t*-test was used to measure the association between mutagen sensitivity and p53 overexpression or telomerase activity. The odds ratio (OR) (Woolf, 1955) was also used as a measure of the strength of association between telomerase activity and p53 overexpression. Logistic regression was conducted to estimate risks, which were adjusted for multiple factors by using STATA statistical software and SAS.

### RESULTS

Characteristics of the lung cancer patients are given in Table 1. Cigarette smoking information was available for 86 patients. Ten patients (11.6%) had never smoked. Family cancer history information was available for 89 patients, of whom 52 (58.4%) had family history of cancer (23 with lung cancer). Tumour stage information was missing for six patients.

p53 protein overexpression was detected in 63 of 100 lung tumour samples (63%), but was undetectable in 89 of the 91 adjacent normal tissues (98%). p53 overexpression was detected in both early- and late-stage tumours: 24 of 39 stage I tumours (61.5%), seven of 12 stage II tumours (58.3%), 18 of 33 stage II tumours (54.6%), and ten of ten stage IV tumours (100%) had detectable p53 protein (Table 2).

By the highly sensitive TRAP assay with an internal control, 80 of 100 lung tumour samples showed telomerase activity (Table 1)

© Cancer Research Campaign 1999 

**Table 2** Molecular markers by characteristics of the lung cancer patients

| Tumour stages | p53 Positive | p53 Negative | *P*-value | Telomerase activity Positive | Telomerase activity Negative | *P*-value |
|---|---|---|---|---|---|---|
| 1 | 24 (60) | 16 (40) | | 29 (74.4) | 10 (25.6) | |
| 2 | 7 (58.3) | 5 (41.7) | | 12 (100.0) | 0 (0.0) | |
| 3 | 18 (54.6) | 15 (45.4) | | 25 (75.8) | 8 (24.2) | |
| 4 | 10 (100.0) | 0 (0.0) | 0.070 | 9 (90.0) | 1 (10.0) | 0.193 |

**Table 3** Level of p53 expression, telomerase activity and constitutional genetic instability

| | Bleomycin-induced mutagen sensitivity | | | |
|---|---|---|---|---|
| | *n* | Mean breaks/cell | s.d. | *P* |
| p53 overexpression | | | | |
| Positive | 25 | 0.92 | 0.49 | 0.046 |
| Negative | 19 | 0.65 | 0.34 | |
| Telomerase activity | | | | |
| Positive | 36 | 0.83 | 0.46 | 0.391 |
| Negative | 8 | 0.68 | 0.41 | |

to varying degrees. Of the 91 normal specimens, only seven (7.7%) were positive. We also observed that the level of telomerase activity varied dramatically in different individuals (Figure 1 A,B). There were no significant differences in telomerase activity in terms of patients' tumour stage (Table 2).

p53 overexpression was detected in 56 of 80 lung tumour tissue samples (70%) with positive telomerase activity, but only seven out of 20 without telomerase activity (35%) ($P < 0.01$). In Kendall's correlation test, p53 overexpression was significantly correlated with telomerase activity ($P < 0.005$) with a Kendall's Tau-b of 0.290. By univariate analysis, p53 overexpression yielded an OR of 4.3 (95% confidence interval, 1.5–12.2) for telomerase activity. After adjustment by age, sex, ethnicity, smoking status and family history of lung cancer, the OR by p53 overexpression for telomerase activity was 6.7 (95% confidence interval, 1.7–27.7). Figure 1C shows the different levels of p53 protein expression in various samples.

In a subset of the patients, we also measured mutagen sensitivity based on quantification of bleomycin-induced breaks in short-term cultured lymphocytes as a marker of constitutional genetic instability. We found that p53 protein overexpression in tumour tissues was associated with significantly more breaks per cell in lymphocytes (Table 3). The mean breaks per cell was 0.92 for patients with overexpressed p53 in lung tumour tissues, compared with 0.65 for patients with no detectable p53 expression in lung tumour tissues ($P < 0.05$). The mean breaks per cell for patients with positive telomerase activity in lung tumour tissues was higher than that of patients with undetectable telomerase activity in lung tumour tissues (0.83 vs 0.68). However, the difference was not statistically significant.

### DISCUSSION

In the study reported here, p53 overexpression was commonly elevated in the lung tumour tissues studied, but was rarely detectable in adjacent normal tissues. p53 protein overexpression was present both in early- and late-stage tumours, which is consistent with the report that p53 protein accumulation is an early event in carcinogenesis and persists during metastatic progression (Fontanini et al, 1994).

Similarly, we found that telomerase activity was detected in the majority (80%) of primary lung tumour tissues but in only 7% of adjacent non-cancerous tissues. Telomerase activity was measured by a PCR-based TRAP assay with an internal control, which increased the assay's sensitivity and specificity significantly. Telomerase activity in normal tissue could be due to the presence of a few telomerase-positive tumour cells. That the levels of telomerase activity varied dramatically in different tumour tissues may reflect the ratio of mortal to immortal cells in each tumour. Shay's group have suggested that telomerase activity may be a lung cancer malignancy indicator (Hiyama et al, 1995*a*). However, we did not find a significant association between telomerase activity and tumour stage.

We found that there was a significant association between telomerase activity and aberrant p53 protein overexpression. Kruk and Bohr (1996) suggested that telomerase expression was compromised in cells expressing mutated p53. Lung carcinoma cells and other types of tumour cells expressing wild-type p53 have longer telomeres than cells lacking *p53* or with mutated *p53* mutations (Kruk and Bohr, 1996). Hiyama et al (1995*b*) also found a significant association between alterations in telomeric repeat length and loss of *p53* heterozygosity in lung cancer. Wynford-Thomas and colleagues (Wynford-Thomas et al, 1995) further suggested that wild-type p53 may form part of a system that detects either the loss of telomeres directly or the structural consequences of telomere erosion (or both), and subsequently signals growth arrest in $G_1$. Only cells that lack p53 activity (e.g. by mutation) will be able to pass this barrier. Subsequently, *p53* mutant clones will acquire a wide range of DNA lesions as a consequence of having lost the 'guardian of the genome' checkpoint function of p53, and of having the destabilizing effect of further telomere erosion. Ultimately, those cells will also need to re-express telomerase.

Bleomycin sensitivity has been used as a measure of constitutional genetic instability and one of the cancer susceptibility markers (Hsu et al, 1989; Spitz et al, 1989, 1994; Strom et al, 1995; Wu et al,

 © Cancer Research Campaign 1999

1995*a*, 1995*b*, 1996). We found that p53 aberrant expression was more common in bleomycin-sensitive individuals than in non-sensitive individuals, which was consistent with the findings of Gallo et al 1995). In normal cells, p53 levels are extremely low owing to the very short half-life of the protein. Accumulation of p53 protein within neoplastic cells correlates well with the presence of mis-sense mutations, which is a reflection of the increased stability of the mutated p53 protein compared with its wild-type counterpart (Iggo et al, 1990). Furthermore, p53 is a target of benzo[*a*]pyrene, which is a major constituent of carcinogens in cigarette smoking (Denissenko et al, 1996). A significant association between p53 protein over-expression and tobacco smoking has also been observed (Dosaka-Akita et al, 1994). Therefore, individuals with increased susceptibility to carcinogens after exposure to mutagens such as to tobacco smoke or ionizing radiation are at higher risk for lung cancer, in which one of the most common genetic events is *p53* aberrations. Such differences in genetic susceptibility and in *p53* abnormalities in carcinogen-exposed epithelia might have a major impact on assessment of lung cancer risk.

We also found the mean breaks per cell for patients with positive telomerase activity in lung tumour tissues was higher than that of patients with undetectable telomerase activity in lung tumour tissues (0.83 vs 0.68), although the difference was not statistically significant. We speculated that individuals with constitutional genetic instability may be more prone to mutagen-induced chromosome breakage, which may result in loss of telomeres. The loss of telomeres may further drive genomic instability, which results in chromosome abnormalities and unchecked cell growth.

In summary, our data suggest that telomerase activity is a good tumour marker. In individuals with genetic susceptibility to lung cancer as measured by our mutagen sensitivity assay, p53 over-expression was increased. p53 overexpression, but not telomerase activity, was correlated with bleomycin sensitivity. Excess p53 protein expression was associated with telomerase activation. Therefore, in individuals genetically susceptible to carcinogen exposure, p53 protein overexpression may be common. p53 status may be related to telomerase expression. Telomerase activity may be a later event than p53 overexpression. However, there are some limitations in this study, accumulation of p53 protein within neoplastic cells correlates well with the presence of mis-sense mutations, but we could not detect total deletion, frameshift or non-sense mutations of the *p53* gene that do not result in p53 accumulation (Iggo et al, 1990). Data for telomere length were not available for these tissues. Further study with more extensive characterization of p53 mutations is warranted to confirm and extend our findings.

### ACKNOWLEDGEMENTS

We thank Dr Maureen Goode for her editorial help, Ms Gloria Milton for her secretarial help and Ms Hong Li for her technical support in the manuscript preparation. This work was supported by Grants 1RO3 70191 (XW) and CA 55769 (MRS) from the National Cancer Institute and Devereaux Award from Cancer Research Foundation of America (XW).

### REFERENCES

Denissenko MF, Pao A, Tang M and Pfeifer GP (1996) Preferential formation of benzo[a]pyrene adducts at lung cancer mutational hotspots in p53. *Science* **274**: 430–432

Dosaka-Akita H, Shindoh M, Fujino M, Kinoshita I, Akie K, Katoh M and Kawakami Y (1994) Abnormal p53 expression in human lung cancer is associated with histologic subtypes and patient smoking history. *Anat Pathol* **102**: 660–664

Fontanini G, Vignati S, Bigini D, Merlo GR, Ribecchini A, Angeletti CA, Basolo F, Pingitore R and Bevilacqua G (1994) Human non-small cell lung cancer: p53 protein accumulation is an early event and persists during metastatic progression. *J Pathol* **174**: 23–31

Gallo O, Bianchi S, Giovannucci-Uzzielli ML, Santoro R, Lenzi S, Salimbeni C, Abbruzzese M and Alajmo E (1995) *p53* oncoprotein overexpression correlates with mutagen-induced chromosome fragility in head and neck cancer patients with multiple malignancies. *Br J Cancer* **71**: 1008–1012

Harley CB and Villeponteau B (1995) Telomeres and telomerase in aging and cancer. *Curr Opin Genet Dev* **5**: 249–255

Hiyama K, Hiyama E, Ishioka S, Yamakido M, Inai K, Gazdar AF, Piatyszek MA and Shay JW (1995*a*) Telomerase activity in small-cell and non-small-cell lung cancers. *J Natl Cancer Inst* **87**: 895–902

Hiyama K, Ishioka S, Shirotani Y, Inai K, Himaya E, Murakami I, Isobe T, Inamizu T and Yamakido M (1995*b*) Alterations in telomeric repeat length in lung cancer are associated with loss of heterozygosity in *p53* and Rb. *Oncogene* **10**: 937–944

Hollstein M, Sidransky D, Vogelstein B and Harris C (1991) *p53* mutations in human cancer. *Science* **253**: 49–53

Hsu TC (1983) Genetic instability in the human population: a working hypothesis. *Herditas* **98**: 1–11

Hsu TC, Johnston DA, Cherry LM, Ramkisson D, Schantz SP, Jessup JM, Winn RJ, Shirley L and Furlong C (1989) Sensitivity to genotoxic effects of bleomycin in humans: possible relationship to environmental carcinogenesis. *Int J Cancer* **43**: 403–409

Iggo R, Gotter K, Bartek K, Lane D and Harris AL (1990) Increased expression of mutant forms of *p53* oncogen in primary lung cancer. *Lancet* **335**: 675–679

Kim NW, Piatyszek MA, Prowse KR, Harley CB, West MD, Ho PLC, Coviello GM, Wright WE, Weinrich SL and Shay JW (1994) Specific association of human telomerase activity with immortal cells and cancer. *Science* **266**: 2011–2015

Kruk PA and Bohr VA (1996) Relationship between telomeric length and p53 status. Abstract. *Proc Am Assoc Cancer Res* **37**: 560

Marx J (1994) New link found between *p53* and DNA repair. *Science* **266**: 1321–1322

Piatyszek MA, Kim NW, Weinrich SL, Hiyama K, Hiyama E, Wright WE and Shay JW (1995) Detection of telomerase activity in human cells and tumors by a telomeric repeat amplification protocol (TRAP). *Methods Cell Sci* **17**: 1–15

Shay JW, Pereira-Smith OM and Wright WE (1991) A role for both RB and p53 in the regulation of human cellular senescence. *Exp Cell Res* **196**: 33–39

Spitz MR, Fueger JJ, Beddingfield NA, Annegers JF, Hsu TC, Newell GR and Schante SP (1989) Chromosome sensitivity to bleomycin-induced mutagenesis, an independent risk factor for upper aerodigestive tract cancers. *Cancer Res* **49**: 4626–4628

Spitz MR, Hoque A, Trizna Z, Schantz SP, Amos CI, King TM, Bondy ML, Hong WK and Hsu TC (1994) Mutagen sensitivity as a risk factor for second malignant tumors following upper aerodigestive tract malignancies. *J Natl Cancer Inst* **86**: 1681–1684

Strom SS Wu XF, Sigurdson AJ, Hsu TC, Fueger JJ, Lopez J, Tee PG and Spitz MR (1995) Lung cancer, smoking patterns, and mutagen sensitivity in Mexican-Americans. *Monogr Natl Cancer Inst* **18**: 29–33

Woolf B (1955) On estimating the relation between blood group and disease. *Ann Hum Genet* **19**: 251–253

Wright WE, Pereira-Smith OM and Shay JW (1989) Reversible cellular senescence: implications for immortalization of normal human diploid fibroblasts. *Mol Cell Biol* **9**: 3088–3092

Wu XF, Hsu TC, Annegers JF, Amos CI, Fueger JJ and Spitz MR (1995*a*) A case-control study of nonrandom distribution of bleomycin-induced chromatid breaks in lymphocytes of lung cancer patients. *Cancer Res* **55**: 557–561

Wu XF, Delclos GL, Annegers FJ, Bondy ML, Honn SE, Henry B, et al (1995*b*) A case-control study of wood-dust exposure, mutagen sensitivity, and lung-cancer risk. *Cancer Epidemiol Biomarkers Prev* **4**: 583–588

Wu XF, Hsu TC and Spitz MR (1996) Mutagen sensitivity exhibits a dose-response relationship in case-control studies. *Cancer Epidemiol Biomarkers Prev* **5**: 577–578

Wynford-Thomas D, Bond JA, Wyllie FS and Jones CJ (1995) Does telomere shortening drive selection for *p53* mutation in human cancer? *Mol Carcinog* **12**: 119–123

Zhang W, Grasso L, McClain CD, Gambel AM, Cha Y, Traveli S, Diesseroth AB and Mercer WE (1995) *p53*, independent induction of WAFI/Cip1 in human leukemia cells is correlated with growth arrest accompanying monocyte/macrophage differentiation. *Cancer Res* **55**: 668–674

© Cancer Research Campaign 1999
